# Optimization of fluidized bed drying process parameters and quality evaluation of ready to use onion slices

**DOI:** 10.1038/s41598-025-25036-x

**Published:** 2025-11-10

**Authors:** Shahzad Faisal, Arjumand Yousuf, Bilal Ah. Langoo, Sadhna Mishra, Mohammad Muzamil, Mudasir Ali, Rishi Richa, Vijay Kumar, Javed Masood Khan, Per Erik Joakim Saris, Arun Kumar Gupta, Bindu Naik

**Affiliations:** 1 College of Agricultural Engineering & Technology, Sher-e-Kashmir, University of Agricultural Sciences and Technology, Kashmir, 190025 J&K India; 2 Faculty of Agricultural Sciences, GLA University, Mathura, 281406 India; 3 School of Biosciences, Swami Rama Himalayan University, Jolly Grant, Dehradun, 248016 Uttarakhand India; 4 Department of Food Science and Nutrition, College of Food and Agricultural Sciences, King Saud University, 2460, Riyadh, 11451 Saudi Arabia; 5 Department of Microbiology, Faculty of Agriculture and Forestry, University of Helsinki, Helsinki, Finland; 6 Department of Food Processing Technology, College of Post-Harvest Technology and Food Processing, Sardar Vallabhbhai Patel University of Agriculture and Technology, Modipuram, Meerut India; 7 Department of Food Science and Technology, Graphic Era Deemed to be University, Bell Road, Clement Town, Dehradun, 248002 Uttarakhand India; 8 School of Agriculture, Graphic Era Hill University, Clement Town, Dehradun, Uttarakhand India

**Keywords:** Onion drying, Temperature, NaCl concentration, Drying characteristics, Optimization, Page’s model sensory attributes, Biochemistry, Biotechnology

## Abstract

This study investigates the kinetics and optimization of fluidized bed drying of onion slices using CCRD design by Response Surface Methodology, aiming to enhance drying efficiency and preserve product quality. The effects of drying process parameters viz., drying temperature (43.18, 50,60,70, & 76.82 °C), NaCl concentration (6.59, 10, 15, 20, & 23.41%) and bed thickness (1.636, 3,5, 7, & 8.362 mm) on dehydration ratio, rehydration ratio, color change, ascorbic acid content and overall acceptability were evaluated using multiple response optimization techniques. Optimization analysis revealed that a drying temperature of 70 °C, NaCl concentration of 20%, and bed thickness of 3 mm yielded the highest desirability value of 0.637. Under these optimized conditions, the dehydration ratio, rehydration ratio, color change, ascorbic acid content, and overall acceptability of onion slices were measured at 6.76, 5.87, 4.85, 8.06 and 4.02, respectively. Higher drying temperatures, NaCl concentrations and lower bed thickness were associated with faster drying rates. Various mathematical models, including Page, Logarithmic, Henderson and Pabis, Midilli and Newton models, were employed to characterize the drying kinetics, with the Page Model demonstrating superior fit to experimental data. Furthermore, quality parameters such as moisture content, water activity, ascorbic acid content, color change and crispiness of onion slices stored for three months under optimized conditions and packaged in different materials (LDPE and HDPE) were evaluated. Results indicate that the optimized fluidized bed drying process parameters effectively maintained the quality of onion slices during storage.

**Supplementary Information:**

The online version contains supplementary material available at 10.1038/s41598-025-25036-x.

## Introduction

 Onion (*Allium cepa* L) holds a prominent position in the realm of vegetable cultivation, esteemed for both its culinary significance and nutritional value. Belonging to the Liliaceae family within the Plantae kingdom, onions have been cultivated for millennia, with historical roots tracing back to regions such as Northwest India, Afghanistan, the former Soviet Union, and China^1^. Presently, global production of onions exceeds 700 lakh metric tonnes, with China and India leading in production, contributing over 25% and 20% of the total yield, respectively^2^. In India, Maharashtra, Madhya Pradesh, and Karnataka emerge as the primary onion-producing states, collectively contributing over 50% of the nation’s onion yield. Even in northern regions like Jammu and Kashmir, onions are cultivated, highlighting the crop’s ubiquity and economic significance. With a rich nutrient profile comprising moisture, carbohydrates, proteins, vitamins, and minerals, fresh onions are integral to culinary traditions worldwide^3^.

Beyond their gastronomic appeal, onions harbor medicinal properties attributed to compounds such as allyl-propyl-disulphide, offering hypocholesterolemic, thrombolytic, and antioxidant effects^4^. However, despite their esteemed status, onions are inherently perishable, with their moisture content rendering them susceptible to spoilage during storage. Factors like mechanical handling, enzymatic reactions, and microbial activity accelerate deterioration, posing challenges for growers and consumers alike. Furthermore, the seasonal nature of onion production coupled with fluctuating demand results in market imbalances, often reflected in volatile prices ranging from Rs. 100 to Rs. 150 per kg in India. Such instability perpetuates significant post-harvest losses, estimated at 40–45%, encompassing sprouting, rotting, and physical degradation^5^. To mitigate these losses and stabilize the market, efficient preservation techniques are imperative. Among these techniques, drying emerges as a promising avenue, offering extended shelf life, reduced volume, and convenient storage. Tray drying or tunnel air-drying methods are used for drying of the fruits and vegetables. The onions are generally dried by air-drying methods but fluidized dryer is efficient than tray and cabinet dryer^6,7^.

Specifically, fluidized bed drying presents itself as a viable method, particularly suitable for heat-sensitive materials like onions. This approach facilitates efficient heat and mass transfer, resulting in high-quality dried products within a shorter timeframe^8,9^. While research into onion dehydration worldwide has yielded significant insights, a comprehensive understanding of how various factors affect the quality attributes of onions dried in a fluidized bed remains elusive. Therefore, this study aims to bridge this gap by investigating the fluidized bed drying of onions with specific objectives. Firstly, the study seeks to scrutinize how various drying process variables influence the fluidized bed drying characteristics of onions slice. Secondly, it endeavors to develop drying kinetics models and optimize drying process parameters to enhance efficiency and maintain or improve the quality of the dried onions for 3 months storage period. Although various advanced drying techniques such as microwave, infrared, and freeze drying have been explored for fruits and vegetables, their practical scalability, energy efficiency, and cost-effectiveness remain limiting factors for widespread adoption, especially in small and medium-scale processing units. Fluidized bed drying, by contrast, offers a balance between technological advancement and operational feasibility, making it one of the most preferred drying techniques in industrial practice due to its uniform drying, shorter drying time, and better control over product quality. The novelty of the present investigation lies in its comprehensive and industrially relevant approach. It focuses on developing ready-to-use chopped onion slices suitable for off-season use in vegetables, saag, and gravies—an area rarely addressed in existing research. Unlike earlier studies that mainly concentrate on drying kinetics or single-process optimization, this study holistically integrates drying kinetics modeling, process parameters optimization using response surface methodology (RSM), and a three-month storage stability evaluation using two commercial packaging materials (HDPE and LDPE). Key quality parameters such as dehydration ratio, rehydration ratio, color change, ascorbic acid content, water activity, and sensory attributes were assessed. Additionally, sodium chloride (NaCl) concentration was optimized alongside drying temperature and bed thickness to evaluate its role in enhancing drying performance and preserving quality. This integrated framework not only enhances process efficiency but also aligns with consumer demand for convenience-oriented, shelf-stable products. Few previous studies have combined drying, packaging, and usability assessment in a single experimental design, highlighting the uniqueness and industrial potential of this work.

## Materials and methods

### Raw material

Fresh Onion, well-graded, and good quality Nasik red onions were procured from the local market of Shalimar. The onions were brought to the Food Processing Laboratory, College of Agricultural Engineering and Technology, SKUAST-Kashmir, Shalimar. Care was taken to prevent any visible mechanical damage, rooting, sprouting, and microbial infestation.

### Sample preparation

The onions were manually peeled using stainless steel knives. These were cut into equal halves longitudinally and then onion slices of 4 × 4 mm thickness were cut with utmost precision transversely. The main purpose of slicing was to ensure a uniform shape and size throughout the experiment.

### Pre-treatment

An osmotic solution of water and sodium chloride (NaCl) was prepared as per the treatment combinations to dip onion slices into it by maintaining a solid to syrup ratio of 1:5 for 10 minutes^10^. Prior to drying, water adhered to the surfaces of onion samples pre-treated with NaCl solution was drained and removed with tissue paper. NaCl was selected as a pre-treatment as it prevents color change during drying and has no adverse effects on health.

### Experimental design

The three independent variables of study viz. drying air temperature, NaCl concentration, and bed thickness were selected for onion slice drying. The five levels of each independent variables were decided through CCRD showed in Table 1 with coded and actual value. The RSM (Response Surface Methodology) was used to decrease the number of experimental runs without affecting the accuracy of the findings and to evaluate the interaction effects of process variables on the response variables^11^. The combinations of process variable levels in each experiment were decided by the central composite rotatable design (CCRD). The plan consisted of total 20 experiments in each group with the first 8 experiments in the first order part (factorial part); next 6 experiments in the second order part (axial part) and the remaining 6 experiments at the central point (Table 2). Three factorial point levels (−1,0,+1) were decided through literature and preliminary experiments on fluidized bed dryer, whereas the two axial point levels (-α (−1.682 and + α (+ 1.682) of coded value of independent variables (drying temperature, NaCl concentration and bed thickness**)** were computed for actual value as follows:

$$\text{Coded value} = \frac{\text{Actual value-Centre point}}{\text{Interval}}$$

**Table 1:** Process variables and their levels.

Process Variables	Coded Levels of Treatment				
	−1.682 (-α)	−1	0	+ 1	+ 1.682 (+α)
**Name**	**Actual levels of Treatments**				
**Drying Temperature (** ^**o**^ **C)**	43.18	50	60	70	76.82
**NaCl concentration (%)**	6.59	10	15	20	23.41
**Bed Thickness(mm)**	1.636	3	5	7	8.362

Total number of experimental runs for three independent variables was decided for central composite design as follows:

**Factorial point experiments** = 2^k^ = 23 = 8

**Axial point experiments** = 2k = 2 × 3 = 6

where,

k = No. of independent variables.

**Centre point experiments** = N_o_= 6 (chosen) (should be ≥ 5).

Total no. of experiments = 2^k^ + 2k + N_0_ = 8 + 6 + 6 = 20

Dehydration ratio, rehydration ratio, color change, ascorbic acid and sensory were evaluated as response. For any dried product (fruits and vegetables) dehydration, rehydration ratio and color change are key response variables to analyse the quality of dried products as well as its consumer acceptability. Dehydration ratio gives an idea about the raw material requirement to obtain a unit quantity of dried product. Whereas rehydration ratio tells us about the extent of shrinkage and case hardening during the drying process which affect the quality of end product significantly. Colour change during drying is most important factor for appearance and acceptability of dried product influenced by the salt concentration used for pre-treatment. It is also seen from Table 1 Practically, it is not feasible to maintain a bed thickness of exactly 1.636 mm; therefore, the value was rounded to two decimal places.

**Table 2:** Experimental runs for central composite rotatable design.

No. of exp.	Coded values		
	Temperature (^o^C)	NaCl Concentration (%)	Bed thickness (mm)
8	± 1	± 1	± 1
2	± 1.682	0	0
2	0	± 1.682	0
2	0	0	± 1.682
6	0	0	0
**Total = 20**			

### Experimental setup

A laboratory fluidized bed dryer supplementary Fig. S1 was used for experimental purposes. It consisted of a temperature controller, an electric heater, and an air blower powered by a 1.5 kW electric single-phase motor. The air heating system provided an air temperature of up to 110 °C for the entire operating range with an accuracy of ± 1 °C. Air flow was regulated using a knob, ensuring uniform drying and fluidization.

### Experimental procedure

**Fig. 1:**
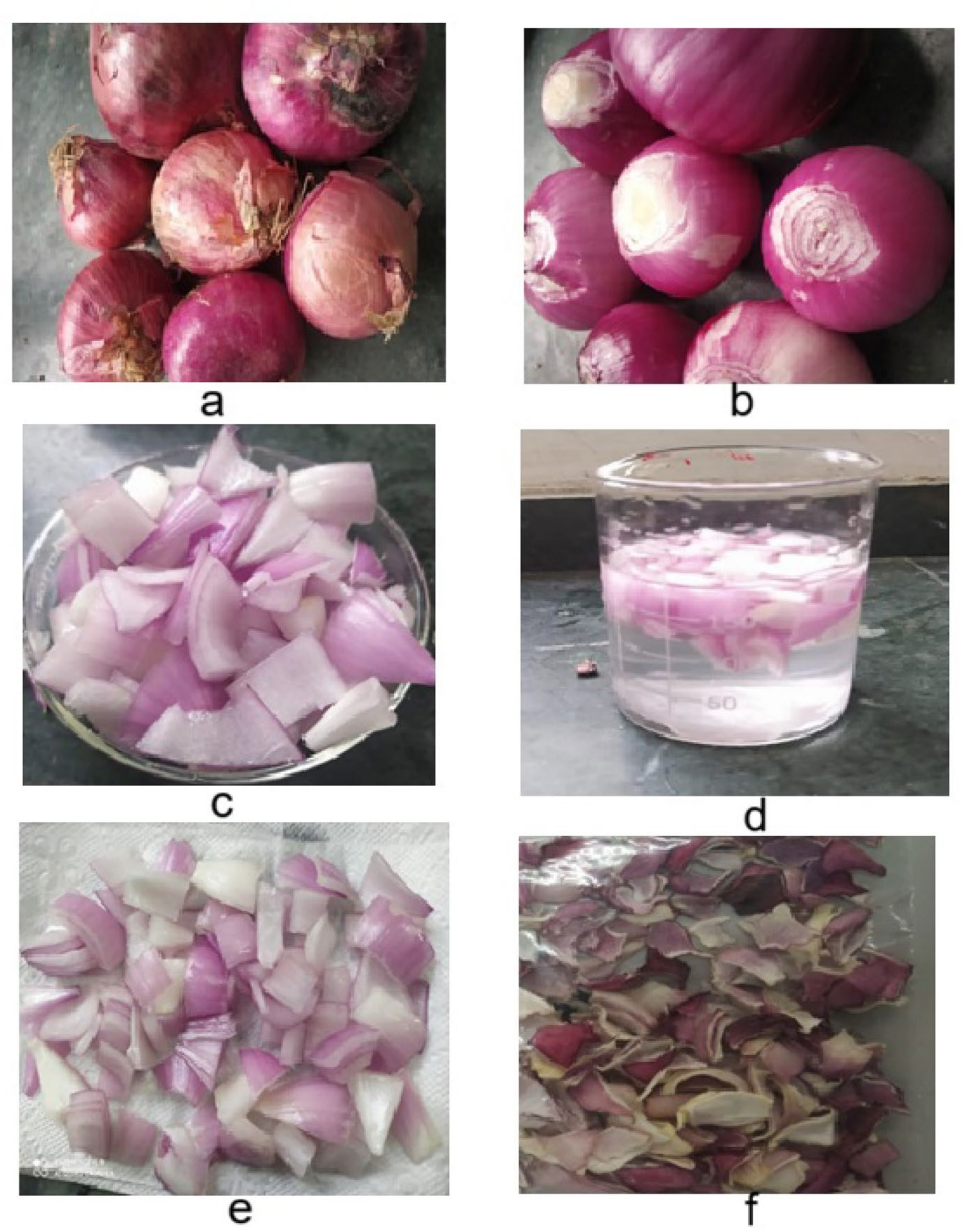
Different stages of onion slices processing from raw material to dried product; **a**- Fresh onion; **b**-peeled onion; **c**- onion slices; **d**-Pretreatment; **d**- pretreated onion slices; **e**-dried onion slices.

Onion slices pre-treated with NaCl solution (6.59, 10, 15, 20 and 23.41%) were dried in a fluidized bed dryer at varying temperatures (43.18 (≈ 43), 50,60,70 and 76.82 °C (≈ 77), and bed thicknesses (1.636, 3,5 and 8.362 mm). Samples were drawn at regular intervals for weighing until the moisture content was reduced below 10% d.b. The dried onions were cooled, packed in polythene bags, and stored for further analysis such as Dehydration ratio, rehydration ratio, color change, ascorbic acid and sensory as shown in Fig. 1.

### Drying characteristics

Drying characteristics were evaluated through curves such as Moisture content vs. time of drying, drying rate vs. time of drying, and Moisture ratio vs. time of drying, revealing the mechanism of moisture migration and heat and mass transfer phenomena. Drying Parameters Moisture content, moisture ratio and equilibrium moisture content were determined using standard methods. The rate of drying was calculated and various drying models were fitted to the experimental data to discuss the drying kinetics. Determination of Response Variables dehydration ratio, rehydration ratio, color change, ascorbic acid content, and mean sensory score of dried onion were determined using standard procedures. Optimum drying parameters numerical optimization of response variables was carried out through RSM and graphical optimization of process variables was performed using contour plots to select the range of variables without affecting the response value.

### Determination of moisture content

Initially the moisture content (m.c % wb) of onion samples were determined by using infra-red moisture meter, made by Toshibha India Co. Infra-red moisture meter used, provided the value of the moisture content directly on a wet basis, was a semi-automatic moisture meter. In experiments, the loss of weight of samples was determined using digital balance. For the calculation of moisture content, the weight difference was changed into corresponding moisture loss. The moisture content was estimated using the following equation^12^ :

$$\text{Moisture content} (\%\text{db}) = \frac{\text{W}_1-\text{W}_2}{\text{W}_2} \times 100$$

Where,

W_1_ = Initial weight of the sample (g)

W_2_ = Bone dry weight of the sample (g)

### Determination of moisture ratio

Moisture ratio was determined using the following equation.

$$\text{MR} = \frac{\text{M}-\text{M}_\text{e}}{\text{M}_\text{o}-\text{M}_\text{e}} \times100$$

Where,

M = Moisture content of sample at any time of drying (%db).

M_e_ = Equilibrium moisture content attained (%db).

M_o_ = Initial moisture content of sample (%db).

### Determination of drying rate

The rate of drying was calculated by the decrease in moisture content (%db) of the onion samples per unit time (min), as determined by the following equation^12^.

$$\frac{\text{dM}_\text{i}}{\text{dT}_\text{i}} = \frac{\text{M}_\text{i}-\text{M}_{\text{i}+1}}{\text{t}_{\text{i}+1} - \text{t}_\text{i}}$$

Where,

$\frac{\text{dM}_\text{i}}{\text{dT}_\text{i}}$ = Drying rate, loss of moisture per min, at i^th^ drying time (%db)

M_i_ = Moisture content at i^th^ time interval

### Drying kinetics modelling

The experimental data was fitted in different drying models to see their feasibility to characterize the drying behaviour of onion slice (Table 3). Following drying models were fitted through linear and non-linear regression technique using NLREG software (6.2 version) to check their feasibility to explain the drying kinetics of onion slices on the basis of their associated R^2^ (coefficient of determination) and SEE (standard error of estimate)^12,13^.

**Table 3:** Drying model for drying kinetics modelling.

S.No.	Model name	Model Equation	Reference
1.	Newton	MR= exp(-kt)	^14^
2.	Page’s	MR = exp(-kt^n^)	^15^
3.	Logarithmic	MR = a exp(-kt^n^) + b	^16^
4.	Midilli	MR = a exp(-kt^n^) + bt	^17^
5.	Handerson and Pabis	MR = a exp(-kt^n^)	^18^

Where:

MR = Moisture ratio.

T = Drying time (min);

K = empirical coefficients in the drying model (min^−1^);

a, b = empirical constants in drying model.

N = consistency index

### Numerical optimization of process variables

Numerical optimization of response variables was carried out through RSM (Response surface methodology), using the commercial statistical package “Design Expert Software version 13.0 State-Ease Inc., Minneapolis, USA”. The variables of the study viz. drying air temperature, NaCl concentration and bed thickness were optimized by compromising the independent variables and responses for their acceptability by setting goal in target, maximize and minimize as per desirability. To validate the optimal results, three confirmation experiments with recommended optimal conditions were carried out. The variation percentage between the observed and predicted value is determined by following formula.

$$\:variation\:\%=\:\frac{|Predicted\:value-Actual\:value|}{Predicted\:value}X\:100$$

### Storage study of optimized dried onion slice

The onions dried under optimized fluidized bed drying conditions were packed and sealed in different packaging materials LDPE (200gauge) and HDPE (200 gauge).The shelf life of dried onion slices was evaluated for the period of three (03) months as per the intervals, under ambient conditions Table 4. The packages were opened at each storage interval for quality evaluation according to the plan of storage studies as follows:

**Table 4:** Experimental plan for shelf life determination during storage study.

Variables	Levels	Responses
**Packaging material**	**Two types of packages**	a) Water activity b) Moisture content c) Ascorbic acid (mg/100 g) d) Colour change (ΔE) e) Crispiness
	• HDPE • LDPE	
**Storage period (days**	**Three months of storage** • 0 • 15 • 30 • 45 • 60 • 70 • 80 • 90	

Experimental design = Factorial CRD

Total experiments = 2 × 8 × 5 × 3replications = 240

### Determination of water activity

The water activity of the optimized dried onion sample was calculated by using water activity meter (PRE AQUA LAB, Water activity analyser, SN: PRE000197).

### Determination of crispiness

The crispiness of the dried onion was determined using texture analyser (TA.HD. plus texture analyser). A spherical probe of 6.35 mm diameter was used to fracture the slices. A trigger force of 25 g, a crosshead pre-test speed of 10 mm/s, a test speed of 5 mm/s and a post test speed of 10 mm/s were used as instrumental settings. The number of force peaks in a time versus force curve was obtained during the fracturing of the onion slices to determine the crispiness.

### Determination of total plate count (cfu/g)

Microbial analysis was ascertained by total plate count (TPC) after 90 days of storage. The method used for microbial analysis was serial dilution and pour plate method as outlined by Ranganna, 1986.10 g of dried onion sample was mixed in 100 ml sterilized water blank to give 10^−1^ dilution and subsequent dilutions were made upto 10^−6^ by transferring serially 1 ml of the dilution into 9 ml sterilized water blanks. The total plate count was estimated by transferring 1 ml of dilutions to sterilized petri dishes and poured with PCA (Plate Count Agar) and plates were kept for incubation at 36 ± 1 °C for 48 h. Calculation and recording of colony growth was carried out in colony forming units per gram (cfu/g).

## Results and discussion

### Drying characteristics of onion slices

**Fig. 2:**
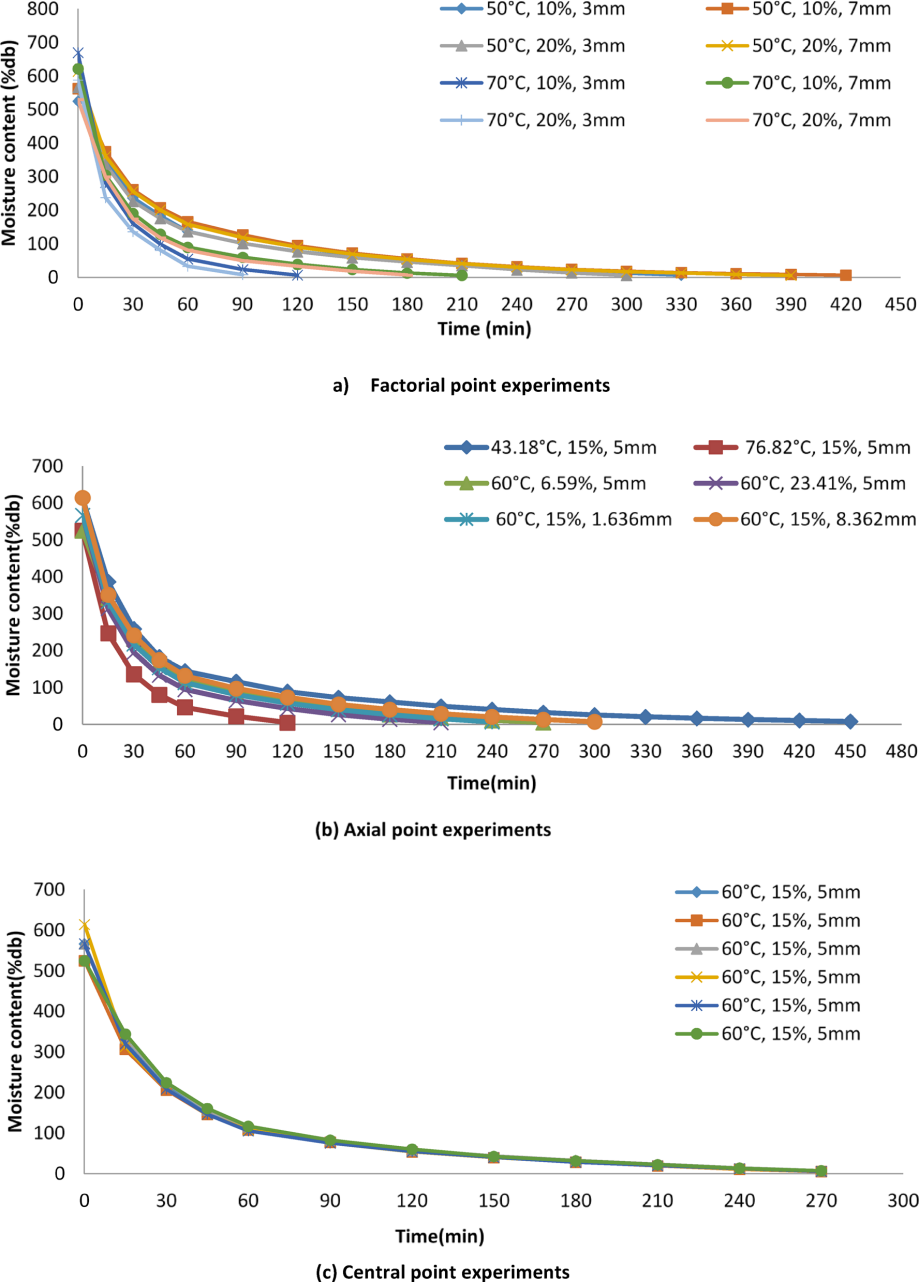
Variation of the moisture content (%db) with drying time (min) at different temperature, NaCl concentration and bed thickness at factorial, axial and central points.

The drying behavior of onion slices was examined across various drying temperatures (43.18 °C, 50 °C, 60 °C, 70 °C, and 76.82 °C), NaCl concentrations (6.59%, 10%, 15%, 20%, and 23.14%), and bed thicknesses (1.636 mm, 3 mm, 5 mm, 7 mm, and 8.362 mm) followed a typical falling-rate period, indicating that moisture removal was primarily governed by internal diffusion rather than surface evaporation. As drying temperature increased, drying time decreased notably, which can be attributed to increased thermal driving force and accelerated moisture migration from the interior to the surface. Initially, there was a rapid decrease in moisture content, followed by a slower decline as shown in Fig. 2 (a, b,c). Moisture loss occurred more rapidly at higher temperatures, such as 76.82 °C (+α), compared to lower temperatures like 43.18 °C (–α). The elevated temperature facilitated greater heat transfer, thereby enhancing moisture removal from the onion slices (Fig. 2a, b,c). This phenomenon aligns with findings from the findings of Akpinar and Bicer^19,20^. Furthermore, moisture loss was faster with thinner bed thicknesses, such as 1.636 mm (-α), compared to thicker ones like 8.362 mm (+α). This accelerated moisture loss with reduced bed thickness could be attributed to the increased surface area per unit mass of moisture available. By reducing the bed thickness, the path length for mass transfer decreases, resulting in quicker moisture loss^21^ and^22^also observed similar trends. At a higher NaCl concentration of 23.41% (+α), moisture loss was faster compared to 6.59% (-α). This effect may stem from the osmotic solution’s ability to draw out a significant amount of moisture from the onion slices, thereby reducing the drying time. Comparable observations were made by BobiĆ et al^10^. and Revaskar et al.^23^.

**Fig. 3:**
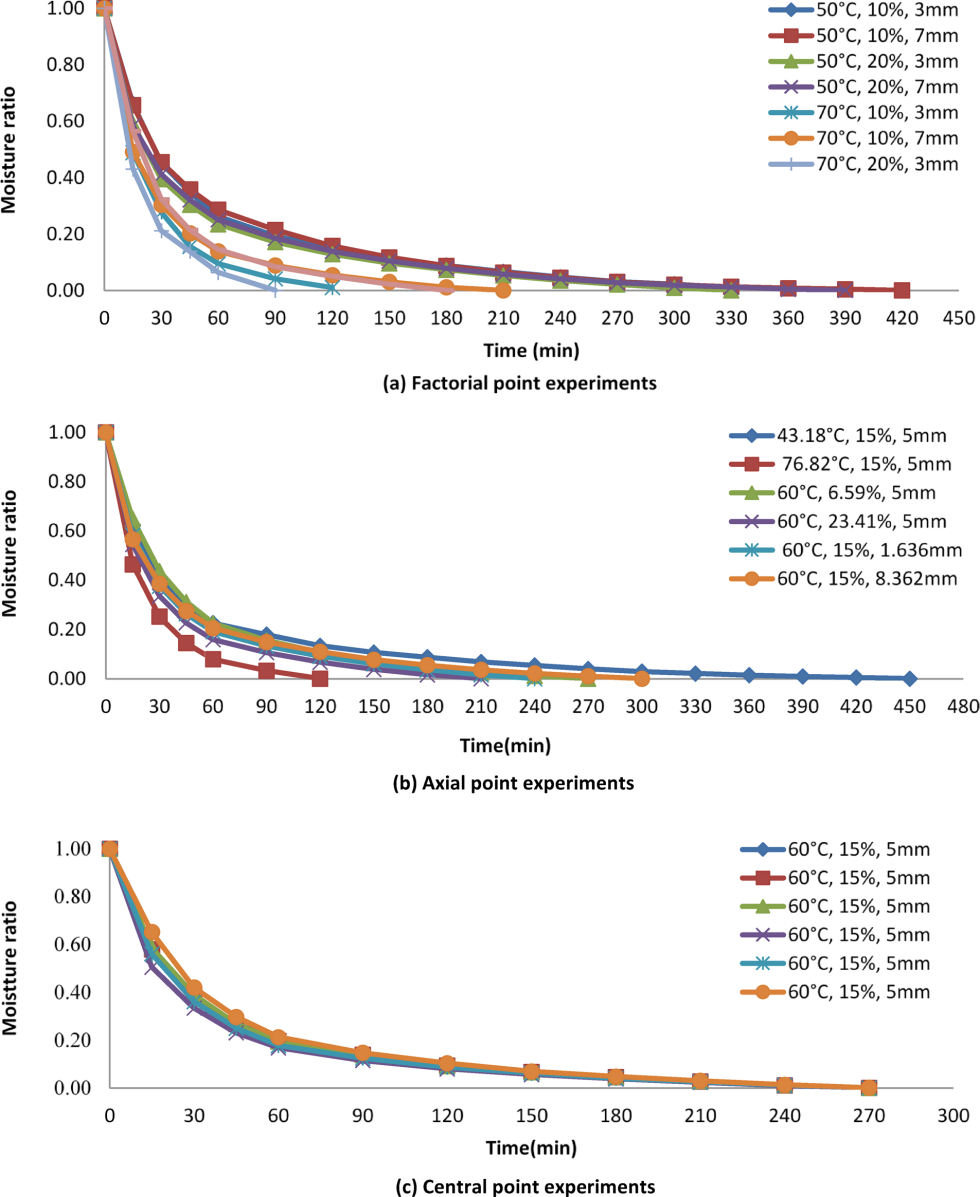
Variation of the moisture ratio with the drying time (min) at different temperatures, NaCl concentrations and bed thickness at factorial, axial and central points.

The moisture ratio curves Fig. 3 (a, b,c) indicate an initial rapid decline in moisture ratio across all experiments, followed by a slower rate of decrease in the later stages of drying. A steeper slope at higher temperatures, indicating faster drying kinetics. For instance, slices dried at 60 °C reached the target moisture level significantly faster than those at 50 °C. Notably, a faster decline was observed at lower bed thickness (1.636 mm, -α) compared to higher bed thickness (8.362 mm, +α), likely due to the increased surface area per unit mass of moisture available. This reduction in bed thickness shortens the path length for mass transfer, facilitating a quicker decrease in moisture ratio (Fig. 3a, b,c). Similar findings were reported by Gupta et al.^21^ and Das et al.^22^. Moreover, the moisture ratio decreased more rapidly at higher temperature (76.82 °C, +α) compared to lower temperature (43.18 °C, -α). This can be attributed to the enhanced drying force for heat transfer at higher temperatures, leading to a faster decrease in moisture ratio in onion slices. This trend aligns with observations made by Giner et al.^8^,Akpinar et al^19^. and Brooks et al.^20^. Furthermore, at a NaCl concentration of 23.41% (+α), the moisture ratio decreased more rapidly compared to 6.59% (-α) NaCl concentration. This effect is likely due to the osmotic solution, which removes a significant amount of moisture from the onion slices, consequently reducing the drying time. Similar results were documented by Hussein et al.^10^and Revaskar et al.^23^.

**Fig. 4:**
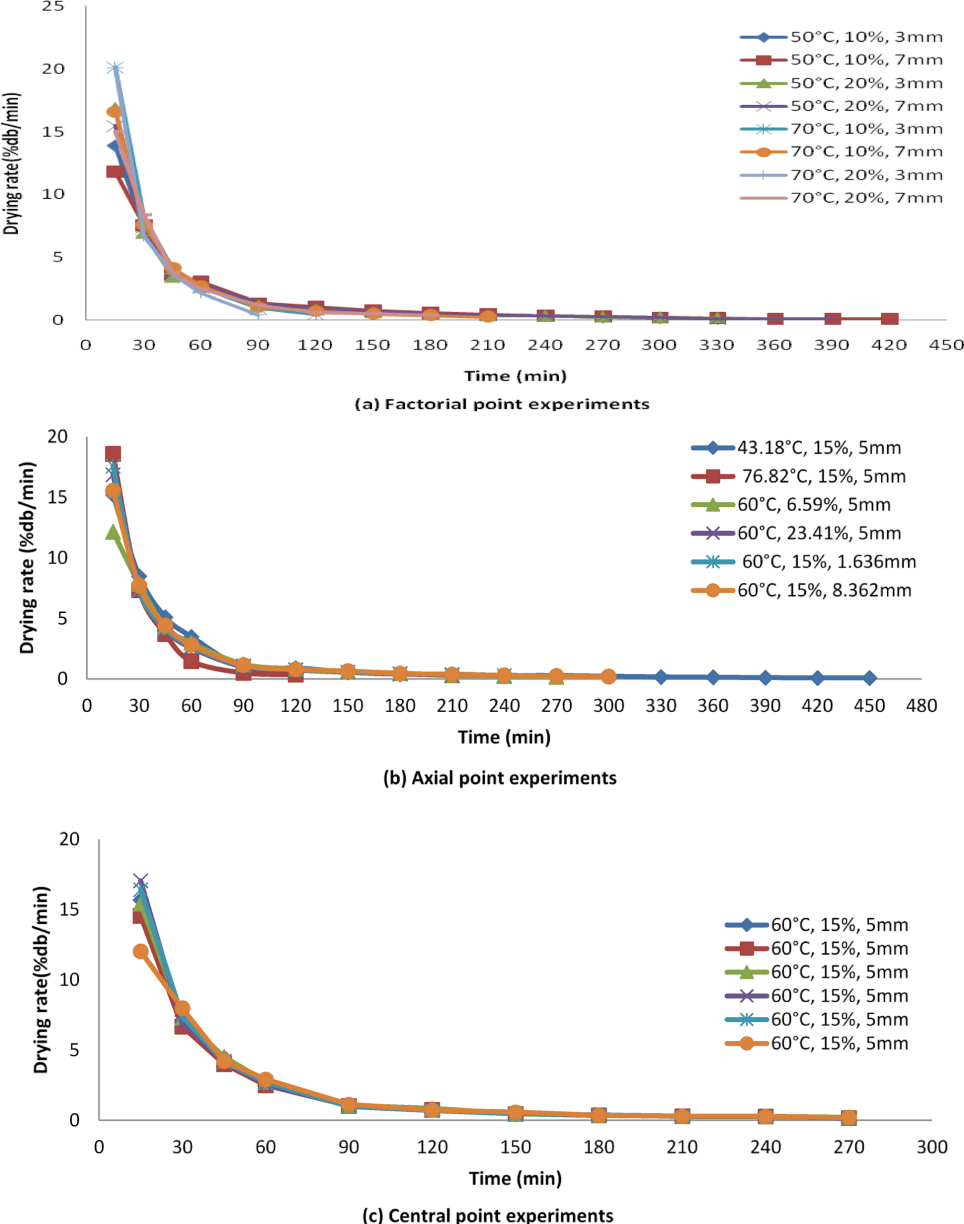
Variation of drying rate (%db/min) with drying time (min) at different temperatures, NaCl concentrations and bed thickness at factorial, axial and central points.

The drying rate exhibited a swifter pace at a lower bed thickness of 1.636 mm (-α) compared to a higher bed thickness of 8.362 mm (+α) shown in Fig. 4 (a, b,c). This acceleration in drying rate resulting from reduced bed thickness may stem from the greater surface area available per unit mass of moisture (refer to Fig. 4a, b,c). Decreasing the bed thickness shortens the path length for mass transfer, thereby enhancing the drying rate. An initial sharp drop followed by a gradual decline, representing a typical falling-rate drying behavior. The maximum drying rate increased with temperature and salt concentration, suggesting that NaCl pre-treatment may have disrupted cell membranes, enhancing internal moisture diffusivity. Similar findings were noted by Gupta et al.^21^ and Das et al.^22^. Likewise, the drying rate proved to be higher at a higher temperature of 76.82 °C (+α) in contrast to a lower temperature of 43.18 °C (-α). The expedited drying rate at higher temperatures is attributed to the rapid removal of moisture, consequently reducing the required drying time. This pattern aligns with observations by^19^and^20^. Furthermore, at a NaCl concentration of 23.41% (+α), the drying rate exhibited swifter progression compared to a concentration of 6.59% (-α). This phenomenon is likely due to the significant removal of moisture from the onion slices facilitated by the osmotic solution, thus shortening the drying time and amplifying the drying rate. Similar observations were reported by Hussein^10^and Revaskar et al.^23^.

### Drying kinetics modelling

The standard error of estimate was found to be the lowest (0.009) and the coefficient of determination (0.9990) was highest in the Page’s model relative to all other four models. Thus, based on the value of R^2^ and SEE, it was found that the page’s model was more satisfactory than the other experimental data models^24^ (Table 5). Page’s model give best result and describe drying characteristics of high moisture content food. The Page model is a semi-empirical modification of the Newton model and introduces an exponent term to better fit experimental drying data. It offers higher flexibility and accuracy, especially in the falling rate period where most moisture removal occurs in onion slice^25^. Onion slice do not dry in a linear fashion due to internal moisture diffusion resistance, cell wall structure, or surface changes. The Page model effectively captures this non-linearity in drying curves. Page’s model was also observed to be best model by different authors for. for onions^23,26^ and tomatoes^10^. The value of consistency index ‘n’ was found to be 0.74 in Page’s model. The value of ‘n’ was close to 1, which is in agreement for high moisture products as indicated in literature^27^.

**Table 5:** Average value of coefficient of determination (R^2^) and standard error of estimate (SEE) of various mathematical models.

S.No.	Model name	SEE	R^2^	Model Constants
1.	Newton	0.0381	0.9808	k = 0.095
2.	Page’s	0.0090	0.9990	k = 0.025,
				*n* = 0.741
3.	Logarithmic	0.0295	0.9708	a = 0.028,
				k = 0.131,
				b = 0.011
4.	Midilli	0.0101	0.9979	a = 0.010,
				k = 0.038,
				*n* = 0.651,
				b = 0.002
5.	Handerson and Pabis	0.0368	0.9840	a = 0.032,
				k = 0.107

### Process optimization for fluidized bed drying of onion

In the fluidized bed drying of onions, three independent parameters were explored: temperature, NaCl concentration, and bed thickness. The focus for process optimization encompassed response variables such as dehydration ratio, rehydration ratio, color change, ascorbic acid content and mean sensory score for overall acceptability.

### Effect of process variables on dehydration ratio

A significant impact of temperature, NaCl concentration and bed thickness on the dehydration ratio of the dried onions. Specifically, the dehydration ratio increased notably with higher temperatures, increased NaCl concentration, and decreased bed thickness. Higher temperatures led to a more pronounced reduction in moisture content as they provided more heat to the onion slices. Additionally, increased NaCl concentration contributed to higher dehydration ratios, possibly due to NaCl acting as an osmotic agent, facilitating faster moisture extraction from the onion slices. Conversely, thicker bed thickness resulted in a decrease in the dehydration ratio, likely because thicker beds led to a slower drying rate, thus extracting moisture at a slower pace Supplementary Table S1 and Supplementary Figs. 2 and 7. These findings align with previous studies by Sahoo et al.^28^.

The second order model was fitted to the experimental data of the dehydration ratio. The effect of drying temperature, NaCl concentration and bed thickness was significant at *P* < 0.05. The quadratic terms and the interaction effect of the drying temperature, NaCl concentration and bed thickness was non-significant (P˃0.05) (Supplementary Table S2). 

### Effect of process variables on rehydration ratio

The impact of temperature, NaCl concentration, and bed thickness on the rehydration ratio of dried onions was found to be significant. A noteworthy increase in the rehydration ratio was observed with higher temperatures, lower NaCl concentrations, and reduced bed thickness. Higher temperatures contributed to a faster drying rate, resulting in less damage to the pore structure of onion slices and consequently enhancing the rehydration ratio. Conversely, greater bed thickness led to increased shrinkage levels due to prolonged heating, thereby reducing rehydration efficiency (Supplementary Table S1, Supplementary Fig S3 and S7). Increased NaCl concentration was associated with a decrease in the rehydration ratio, possibly due to changes in cell permeability caused by osmotic stress, which hindered the cells’ ability to absorb water. Additionally, the salt absorbed during drying might dissolve in water during rehydration, reducing the sample’s weight and consequently the rehydration ratio. Similar observations were reported in previous studies^29–32^.

The second order model was fitted in the experimental data of the rehydration ratio. The effect of drying temperature, NaCl concentration and bed thickness was significant at *P* < 0.05. The interaction effect of the drying temperature, NaCl concentration and bed thickness was non-significant (P˃0.05). In quadratic terms, the effect of bed thickness was non-significant while the impact of drying temperature and NaCl concentration was found to be significant at *P* < 0.05 (Supplementary Table S2).

### Effect of process variables on colour change

Color change exhibited a significant increase with rising temperature, thicker bed thickness, and lower NaCl concentration. The accelerated non-enzymatic browning process at higher temperatures led to enhanced color development in the onion slices. Conversely, higher NaCl concentrations resulted in reduced color change, likely due to the inhibition of the Maillard reaction, consequently impeding enzymatic browning (Supplementary Table S1, Supplementary Fig S4 and S7). The increase in bed thickness correlated with an increase in color change, possibly due to prolonged exposure of onion slices to drying air and extended heating durations. This prolonged exposure could intensify color changes over time. Similar observations were reported by Sutar and Gupta^33^ and Gupta and Shukla^34^.

The second order model was fitted in the experimental data of the colour change. The effect of drying temperature, NaCl concentration and bed thickness was significant at *P* < 0.05. The interaction effect of the drying temperature, NaCl concentration and bed thickness was non significant (P˃0.05). In quadratic terms, the effect of bed thickness and drying temperature was non significant while the impact of NaCl concentration was found to be significant at *P* < 0.05 (Supplementary Table S2).

### Effect of process variables on ascorbic acid

A notable impact of temperature, NaCl concentration, and bed thickness was evident on the ascorbic acid content of dried onions. The ascorbic acid content decreased significantly with increasing temperature and bed thickness, while it increased with higher NaCl concentrations. Elevated temperatures facilitated the degradation of ascorbic acid, with the resultant degradation product, L-dehydroascorbic acid (DHA), contributing to Strecker amino acid degradation, thus forming a browning pigment (Supplementary Table S1). Additionally, as bed thickness increased, the exposure of onion samples to drying air prolonged, leading to a reduction in ascorbic acid content Supplementary Fig S5 and S7. Conversely, higher NaCl concentrations were associated with increased ascorbic acid content, attributed to NaCl’s role as a reducing agent, which prevented the oxidation of ascorbic acid. Similar trends were documented in previous studies^28,35,36^.

The second order model was fitted in the experimental data of the ascorbic acid. The effect of drying temperature, NaCl concentration and bed thickness was significant at *P* < 0.05. The interaction effect of the drying temperature, NaCl concentration and bed thickness was non-significant (P˃0.05). In quadratic terms, the effect of bed thickness and NaCl concentration was non-significant while the impact of drying temperature was found to be significant at *P* < 0.05 (Supplementary Table S2).

### Effect of process variables on sensory score

The mean sensory score for the overall acceptability of dried onions revealed a significant influence of temperature, NaCl concentration, and bed thickness. This score notably decreased with rising temperature and bed thickness, while it increased with higher NaCl concentrations. Among the sensory attributes, color is particularly crucial. The color score values decreased with increasing drying temperature, possibly due to accelerated non-enzymatic browning at higher temperatures (Supplementary Table S1, Supplementary Fig S6 and S7). The aroma of onion slices is predominantly contributed by volatile compounds present in the bulbs, notably the pungent taste resulting from sulphur-containing volatile Allyl-propyl-disulphate oil^37^. The aroma often diminishes with higher drying temperatures, likely due to the loss of volatile onion components. Overall acceptability also declined with increasing bed thickness, potentially due to the extended drying duration required. Conversely, higher NaCl concentrations led to a decrease in overall acceptability, likely because NaCl, acting as a reducing agent, inhibited the enzymatic browning of onion slices. Similar observations were made in previous studies^28,38,39^.

The second order model was fitted in the experimental data of the mean sensory score. The effect of drying temperature, NaCl concentration and bed thickness was significant at *P* < 0.05. The interaction effect of the drying temperature, NaCl concentration and bed thickness was non significant (P˃0.05). In quadratic terms, the impact of all the process variables i.e. temperature, bed thickness and NaCl concentration was significant at *P* < 0.05 (Supplementary Table S2).

### Numerical optimization of process variables

Numerical optimization was carried out by response surface methodology using Design Expert 13.0 software. The main aim of the optimization technique was to determine the best possible combination of the process variables that would result in drying of onion slice with higher acceptable qualities. The goals for responses were set to minimize color change, while to maximize.

Dehydration ratio rehydration ratio, ascorbic acid and mean sensory score Supplementary Table S3. The optimum values of drying air temperature, NaCl concentration and bed thickness were found to be 70 °C, 20% and 3 mm respectively at desirability value of 0.637 (Supplementary Table S3 and S4). At this optimum condition, the predicted values for the dehydration ratio, rehydration ratio, color change, ascorbic acid and mean sensory score were found to be 6.76, 5.87, 4.85, 8.06 and 4.02 respectively (Supplementary Table S3). The actual values of response variables for the onions dried at optimized conditions are used for validation of optimum results obtained (Supplementary Table S4).

### Storage stability of optimized dried onion slices

#### Effect of packaging material and storage days on moisture content

The moisture content of the dried onion sample increased with increase in storage period. Supplementary Table S5 indicates the data pertaining to the impact of different packaging material and storage periods on the moisture content (%db) of dried onion sample. During storage of 90 days there was significant rise in moisture content from 5.770 to 6.725% in dried onion samples. In LDPE pouches, maximum average moisture content of 6.211% was recorded in dried onion samples during 90 days of storage, followed by 6.044% in HDPE pouches.

The onions dried under optimized fluidized bed drying conditions were packed in two different packaging materials (HDPE & LDPE) and were then stored for a period of 3 months under ambient conditions. Packaging plays a significant role on protecting dried food as it acts as a barrier between outside environment and the product inside the packaging material. The quality parameters viz., moisture content, colour change, ascorbic acid, crispiness and water activity were evaluated at the 15 days interval for first 2 months and then at the 10 days interval for last month.

With advancement of storage period there was a gradual rise in moisture content of the dried onion samples irrespective of packaging materials. In LDPE pouches, maximum moisture content was recorded in dried onion samples during 90 days of storage, as compared to HDPE pouches. Since dried onion is highly hygroscopic in nature and can cause moisture gain from air and results in quality loss of dried onion during storage progression. Another possible reason is HDPE prevents the air and moisture gain, due to its low permeability to water vapours and air compared to LDPE pouches hence acts as better packaging material for storage of dried onion^38^. Similar results were reported by Ramya et al.^40^. This increase is primarily due to the higher water vapor transmission rate (WVTR) of LDPE compared to HDPE, which allows more ambient moisture ingress. HDPE packaging, with its superior barrier properties, maintained lower moisture content across storage intervals. Elevated moisture content can compromise texture and accelerate biochemical degradation, underscoring the importance of proper packaging material selection.

### Effect of packaging material and storage days on colour change

The colour change of the dried onion sample increased with increase in storage period. Supplementary Table S5 indicates the data pertaining to the impact of different packaging material and storage periods on the colour change of dried onion sample. During storage of 90 days there was significant increase in colour change from 4.730 to 5.815 in dried onion samples. In LDPE pouches, maximum average colour change of 5.143 was recorded in dried onion samples during 90 days of storage, followed by 5.031 in HDPE pouches.

The colour change of the dried onion sample increased with increase in storage period. In LDPE pouches, maximum colour change was recorded in dried onion samples during 90 days of storage as compared to HDPE pouches. With advancement of storage period there was a gradual increase in colour change of the dried onion samples irrespective of packaging materials, which might be due to non enzymatic browning or **pigment degradation**, likely due to oxidation. The formation of browning pigments due to degradation of sugars during storage might have resulted in browning of dried onions. The lower moisture level of onions inside the HDPE pouches resulted in slower degradation of sugars which might have restricted the non enzymatic browning in them as compared to that of LDPE pouches. HDPE minimized exposure to air and light, resulting in relatively lower color change. Similar findings were observed by Thakur and Gupta^41^. Nonetheless, ΔE values remained within acceptable sensory limits, preserving visual quality throughout the storage period.

### Effect of packaging material and storage days on ascorbic acid

The ascorbic acid content of the dried onion sample decreased with increase in storage period. Supplementary Table S5 indicates the data pertaining to the effect of different packaging material and storage periods on the ascorbic acid of dried onion sample. During storage of 90 days there was significant decrease in ascorbic acid from 7.86 to 6.70 in dried onion samples. In LDPE pouches, minimum average ascorbic acid of 7.36 was recorded in dried onion samples during 90 days of storage, followed by 7.47 in HDPE pouches.

The Ascorbic acid content of the dried onion sample decreased with increase in storage period irrespective of packaging material. In LDPE pouches, minimum ascorbic acid was recorded in dried onion samples during 90 days of storage as compared to HDPE pouches. Significant decrease in ascorbic acid with increase in storage period might be due to the effect of ambient storage temperature on vitamins, and oxidation caused by trapped oxygen inside the packaging material which results in the formation of dehydro ascorbic acid as reported in previous studies^42,43^. Dried onion packed in HDPE pouches experienced least loss of ascorbic acid which may be due to lesser permeability of oxygen in HDPE compared to that of LDPE pouches helping preserve the nutritional quality.

### Effect of packaging material and storage days on water activity

The water activity of the dried onion sample increased with increase in storage period. Supplementary Table S5 indicates the data pertaining to the impact of different packaging material and storage periods on the water activity of dried onion sample. During storage of 90 days there was significant increase in water activity from 0.361 to 0.407 in dried onion samples. In LDPE pouches, maximum average water activity of 0.380 was recorded in dried onion samples during 90 days of storage, followed by 0.374 in HDPE pouches.

The water activity of the dried onion sample increased with increase in storage period. In LDPE pouches, maximum water activity was recorded in dried onion samples during 90 days of storage as compared to HDPE pouches. The increase in the water activity of the samples can be due to hygroscopic nature of the product and ingress of water vapour through the micro cracks and leaks which develop in the packaging material during storage especially in LDPE pouches. Similar results were reported by Mdziniso et al.^44^.

### Effect of packaging material and storage days on crispiness

The crispiness of the dried onion sample decreased with increase in storage period. Supplementary Table S5 indicates the data pertaining to the impact of different packaging material and storage periods on the crispiness of dried onion sample. During storage of 90 days there was significant decrease crispiness from 5.350 to 4.665 in dried onion samples. In LDPE pouches, minimum crispiness of 5.041 was recorded in dried onion samples during 90 days of storage, followed by 5.103 in HDPE pouches.

The crispiness of the dried onion sample decreased with increase in storage period. In LDPE pouches, minimum crispiness was recorded in dried onion samples during 90 days of storage compared to HDPE pouches. With advancement of storage period there was a gradual decrease in crispiness of the dried onion samples irrespective of packaging materials, which might be due to increase in moisture content and water activity. Adsorbed water is supposed to act as a lubricant at high water activities which results in plasticization of polymer chains hence loss of crispiness occurs. At high moisture content, texture becomes soft. This deterioration is linked to moisture reabsorption, which softens the dried tissue structure. HDPE packaging better maintained crispness, as confirmed by sensory scores and physical observations. Although a downward trend was noted, the product retained acceptable crispness for up to three months in both packaging types, validating the feasibility of extended storage. Similar findings were reported previously^45,46^.

### Limitation

Lab scale fluidized bed dryer is used for experimentation, how it will behave at large scale is need to be study. Only NaCl concentration was considered for pre-treatment. Other common treatments (e.g., blanching, use of antioxidants) were not explored and may influence quality outcomes. The storage study was conducted for three months under a single set of environmental condition Other novel drying technology or assisted drying technology such as Infrared and refractive window drying can be used for further drying process to reduce drying time. These limitations can further be analysed for boarder scope and analysis.

## Conclusion

This study on onion slice drying revealed significant impacts of temperature, NaCl concentration and bed thickness on drying characteristics. Higher temperatures, NaCl concentrations and lower bed thickness accelerated drying rates, reducing onion moisture exponentially over time. Page’s model proved most fitting, offering reliable predictions of drying kinetics. Varying conditions affected rehydration and dehydration ratios, color change, ascorbic acid content, and sensory attributes. Temperature elevation increased dehydration and color change but decreased rehydration and ascorbic acid. Higher NaCl concentrations reduced rehydration but boosted ascorbic acid and sensory scores. Conversely, thicker beds increased color change but decreased dehydration, rehydration, and sensory scores. Optimization identified 70 °C, 20% NaCl and 3 mm thickness as ideal, yielding a desirability value of 0.637. Under these conditions, dehydration and rehydration ratios, color change, ascorbic acid, and sensory scores were 6.76, 5.87, 4.85, 8.06 and 4.02, respectively. This study offers practical insights for optimizing onion drying processes to enhance quality, nutrition, and sensory attributes, with implications for industrial applications and further research avenues. as Infrared and refractive window drying can be used for further drying process to reduce drying time. These limitations can further be analysed for boarder scope and analysis. After three months of storage, HDPE pouches were found to be better with higher ascorbic content (7.471), minimum color change (5.031), lower moisture content (6.044), lower water activity (3.74) and maximum crispiness values (5.103) in comparison to LDPE pouches.

## Supplementary Information

Below is the link to the electronic supplementary material.

Supplementary Material 1

## Data Availability

Data will be available on request by Dr. Shahzad Faisal.
