# Supplementary material for: Optimization of fluidized bed drying process parameters and quality evaluation of ready to use onion slices
Source: Sci Rep. 2025 Nov 10;15:39350. doi: 10.1038/s41598-025-25036-x (PMC12603199; doi:10.1038/s41598-025-25036-x)
Supplement: Supplementary file 1 — Supplementary Material 1 [file 41598_2025_25036_MOESM1_ESM.docx]

**Supplementary Files**

**Supplementary Table S1:** Experimental data for the effect of process variables on Responses

| **Expt. No.** | **Coded and Actual values** | | | **Dehydration Ratio** | **Rehydration Ratio** | **Colour Change** | **Ascorbic acid (mg/100 g)** | **Mean Sensory Score** |
| --- | --- | --- | --- | --- | --- | --- | --- | --- |
|  | **Temp.**  **(^o^C)** | **NaCl Conc. (%)** | **Bed thickness (mm)** |  |  |  |  |  |
| 1 | -1 (50) | -1 (10) | -1 (3) | 5.95 | 5.83 | 4.22 | 9.19 | 4.1 |
| 2 | -1 (50) | -1 (10) | 1 (7) | 5.85 | 5.72 | 4.38 | 8.84 | 3.7 |
| 3 | -1 (50) | 1 (20) | -1 (3) | 6.14 | 5.58 | 3.65 | 9.38 | 4.5 |
| 4 | -1 (50) | 1 (20) | 1 (7) | 6.02 | 5.47 | 3.97 | 9.11 | 4.3 |
| 5 | 1 (70) | -1 (10) | -1 (3) | 6.55 | 6.05 | 5.22 | 7.71 | 3.8 |
| 6 | 1 (70) | -1 (10) | 1 (7) | 6.44 | 5.91 | 5.38 | 7.43 | 3.2 |
| 7 | 1 (70) | 1 (20) | -1 (3) | 6.71 | 5.75 | 5.05 | 7.96 | 4.1 |
| 8 | 1 (70) | 1 (20) | 1 (7) | 6.63 | 5.65 | 5.29 | 7.62 | 3.7 |
| 9 | -1.682 (43.18) | 0 (15) | 0 (5) | 5.81 | 5.69 | 3.61 | 9.48 | 4.5 |
| 10 | 1.682 (76.82) | 0 (15) | 0 (5) | 6.85 | 6.42 | 4.98 | 7.53 | 2.9 |
| 11 | 0 (60) | -1.682 (6.59) | 0 (5) | 6.01 | 6.18 | 5.12 | 8.06 | 3.2 |
| 12 | 0 (60) | 1.682 (23.41) | 0 (5) | 6.49 | 5.78 | 4.39 | 8.76 | 4.3 |
| 13 | 0 (60) | 0 (15) | -1.682 (1.636) | 6.45 | 5.59 | 4.19 | 8.61 | 4.4 |
| 14 | 0 (60) | 0 (15) | 1.682 (8.362) | 6.09 | 5.21 | 4.99 | 7.96 | 3.5 |
| 15 | 0 (60) | 0 (15) | 0 (5) | 6.37 | 5.27 | 4.58 | 8.38 | 3.6 |
| 16 | 0 (60) | 0 (15) | 0 (5) | 6.49 | 5.23 | 4.53 | 8.13 | 3.2 |
| 17 | 0 (60) | 0 (15) | 0 (5) | 6.3 | 5.29 | 4.26 | 8.29 | 3.3 |
| 18 | 0 (60) | 0 (15) | 0 (5) | 6.35 | 5.35 | 4.38 | 7.95 | 3.1 |
| 19 | 0 (60) | 0 (15) | 0 (5) | 6.42 | 5.38 | 4.15 | 8.18 | 3.4 |
| 20 | 0 (60) | 0 (15) | 0 (5) | 6.29 | 5.43 | 4.12 | 7.92 | 3.1 |

**Supplementary Table S2:** Analysis of Variance (ANOVA) for the effect of variables on responses

| **Source** | **df** | **P-value** | | | | |
| --- | --- | --- | --- | --- | --- | --- |
|  |  | **DD** | **RR** | **CC** | **AC** | **MSS** |
| Model | 9 | < 0.0001 | < 0.0001 | 0.0001 | < 0.0001 | 0.0004 |
| A-Temperature | 1 | < 0.0001 | 0.0006 | < 0.0001 | < 0.0001 | 0.0002 |
| B-NaCl concentration | 1 | 0.0002 | 0.0015 | 0.0069 | 0.0068 | 0.0011 |
| C-Bed thickness | 1 | 0.0030 | 0.0208 | 0.0121 | 0.0034 | 0.0031 |
| AB | 1 | 0.9610 | 0.8490 | 0.2252 | 0.9668 | 0.7521 |
| AC | 1 | 0.8833 | 0.9494 | 0.8887 | 1.0000 | 0.5307 |
| BC | 1 | 0.9610 | 0.8989 | 0.6757 | 0.9668 | 0.5307 |
| A^2^ | 1 | 0.5186 | < 0.0001 | 0.9501 | 0.0160 | 0.0155 |
| B^2^ | 1 | 0.0531 | < 0.0001 | 0.0095 | 0.0597 | 0.0092 |
| C^2^ | 1 | 0.1001 | 0.8322 | 0.0648 | 0.2929 | 0.0012 |
| Residual | 10 |  |  |  |  |  |
| Lack of Fit | 5 | 0.6278 | 0.1128 | 0.4659 | 0.6768 | 0.3291 |
| Pure Error | 5 |  |  |  |  |  |

**Supplementary Table S3 :** Optimization conditions for different factors and responses

| **Factors/ responses** | **Goal** | **Optimum value** |
| --- | --- | --- |
| Temperature | in range | 70^o^C |
| NaCl Concentration | in range | 20% |
| Bed Thickness | in range | 3mm |
| Dehydration Ratio | Maximize | 6.76 |
| Rehydration Ratio | Maximize | 5.87 |
| Colour Change | Minimize | 4.85 |
| Ascorbic Acid | Maximize | 8.06 |
| Mean sensory score | Maximize | 4.02 |

**Supplementary Table S4:** Validation of optimum results for dried onion slices

| **Parameters** | **Predicted value** | **Actual value** | **Variation** |
| --- | --- | --- | --- |
| Dehydration ratio | 6.76 | 6.645 | 1.70% |
| Rehydration ratio | 5.87 | 5.81 | 1.02% |
| Color change | 4.85 | 4.73 | 2.48% |
| Ascorbic acid | 8.06 | 7.86 | 2.41% |
| Mean sensory score | 4.02 | 3.91 | 2.73% |
| Desirability | 0.637 | | |

**Supplementary Table S5: Effect of packaging material and storage days on the moisture content (%db) of the dried onion slices**

| **Storage days** | **Packaging material** | | **Mean (mc)** | **Packaging material** | | **Mean aw** | **Packaging material** | | **Mean color change** | **Packaging material** | | **Mean ascorbic acid** | **Packaging material** | | **Mean crispiness** |
| --- | --- | --- | --- | --- | --- | --- | --- | --- | --- | --- | --- | --- | --- | --- | --- |
|  | **LDPE** | **HDPE** |  | **LDPE** | **HDPE** |  | **LDPE** | **HDPE** |  | **LDPE** | **HDPE** |  | **LDPE** | **HDPE** |  |
| 0 | 5.770 | 5.770 | 5.770 | 0.361 | 0.361 | 0.361 | 4.730 | 4.730 | 4.730 | 7.860 | 7.860 | 7.860 | 5.350 | 5.350 | 5.350 |
| 15 | 5.830 | 5.810 | 5.820 | 0.363 | 0.362 | 0.363 | 4.770 | 4.760 | 4.765 | 7.800 | 7.810 | 7.805 | 5.290 | 5.310 | 5.300 |
| 30 | 5.910 | 5.860 | 5.885 | 0.367 | 0.365 | 0.366 | 4.830 | 4.810 | 4.820 | 7.720 | 7.750 | 7.735 | 5.227 | 5.260 | 5.244 |
| 45 | 6.020 | 5.937 | 5.978 | 0.371 | 0.368 | 0.370 | 4.920 | 4.880 | 4.900 | 7.600 | 7.660 | 7.630 | 5.140 | 5.190 | 5.165 |
| 60 | 6.170 | 6.037 | 6.103 | 0.377 | 0.372 | 0.374 | 5.050 | 4.980 | 5.015 | 7.427 | 7.530 | 7.478 | 5.030 | 5.110 | 5.070 |
| 70 | 6.370 | 6.157 | 6.263 | 0.386 | 0.378 | 0.382 | 5.260 | 5.130 | 5.195 | 7.190 | 7.340 | 7.265 | 4.910 | 5.014 | 4.962 |
| 80 | 6.637 | 6.310 | 6.473 | 0.399 | 0.386 | 0.393 | 5.570 | 5.340 | 5.455 | 6.873 | 7.080 | 6.977 | 4.770 | 4.870 | 4.820 |
| 90 | 6.980 | 6.470 | 6.725 | 0.417 | 0.398 | 0.407 | 6.010 | 5.620 | 5.815 | 6.460 | 6.740 | 6.600 | 4.610 | 4.720 | 4.665 |
| **Mean (packaging material)** | 6.211 | 6.044 |  | 0.380 | 0.374 |  | 5.143 | 5.031 |  | 7.366 | 7.471 |  | 5.041 | 5.103 |  |
| **CD (p≤0.05)** | Storage days : **0.030**  Packaging material : **0.015**  Storage days × Packaging material: **0.042** | | | Storage days : **0.002**  Packaging material : **0.001**  Days × Packaging material : **0.003** | | | Storage days : **0.028**  Packaging material : **0.014**  Storage days × Packaging material: **0.040** | | | Storage days : **0.025**  Packaging material : **0.013**  Storage days × Packaging material: **0.036** | | | Storage days : **0.028**  Packaging material : **0.014**  Storage days × Packaging material: **0.039** | | |


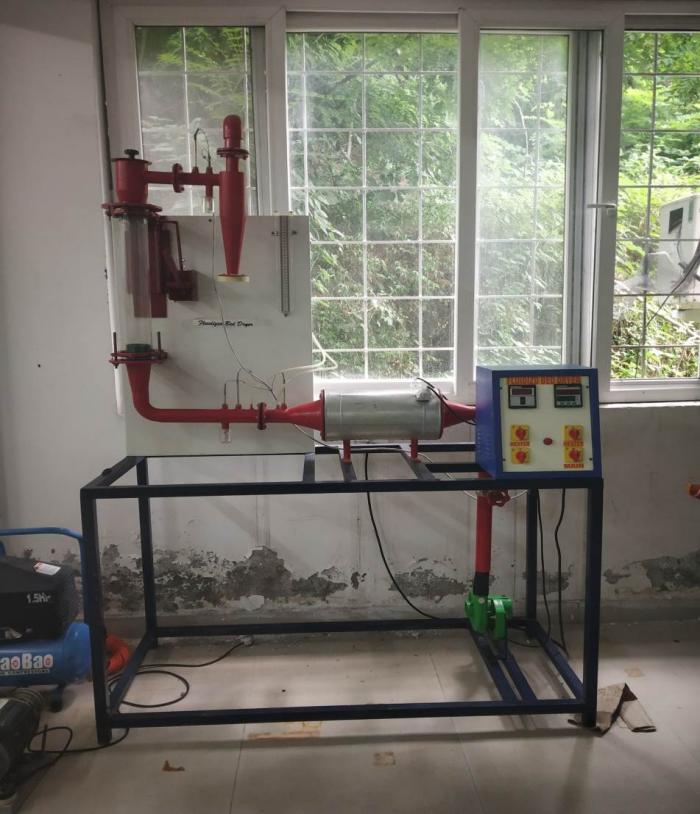


**Fig. S1:** Laboratory scale Fluidized bed dryer used for onion drying

**
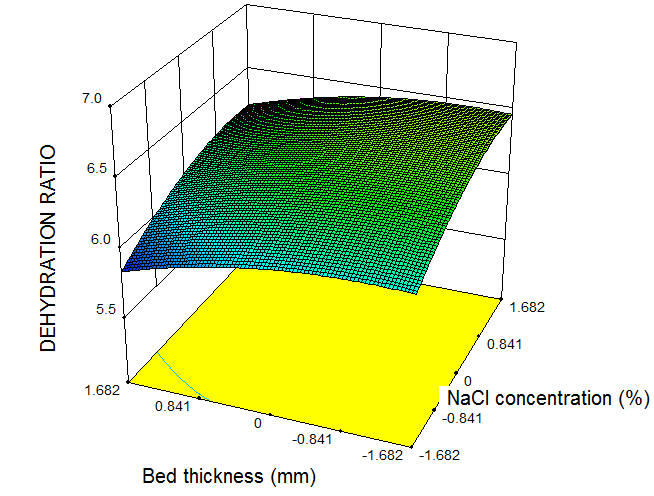
**

**
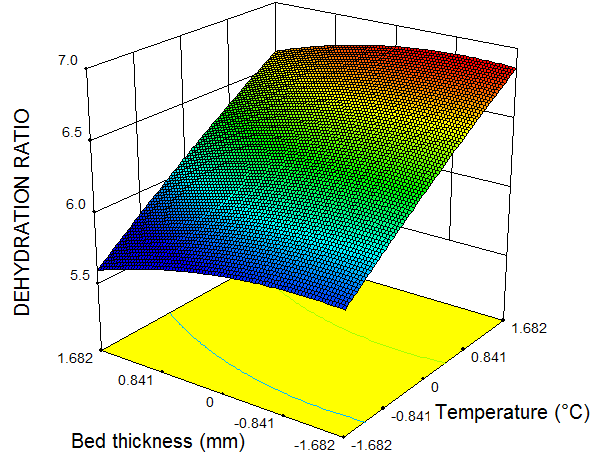
**

**Fig S2:**Response surface plots for the effect of process variables on Dehydration ratio of dried onion slices


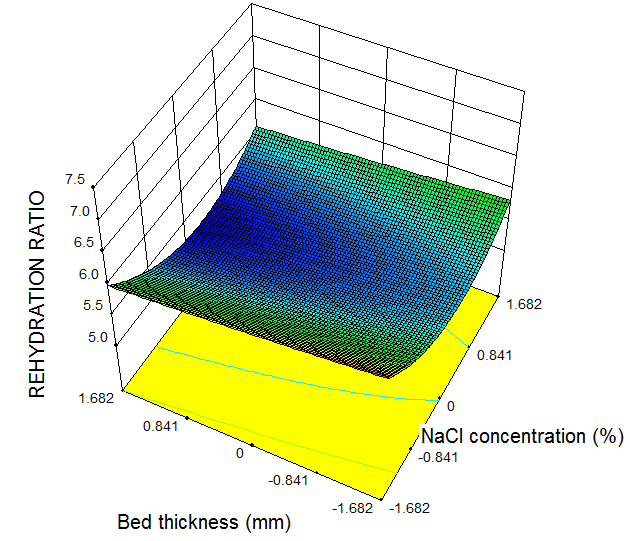

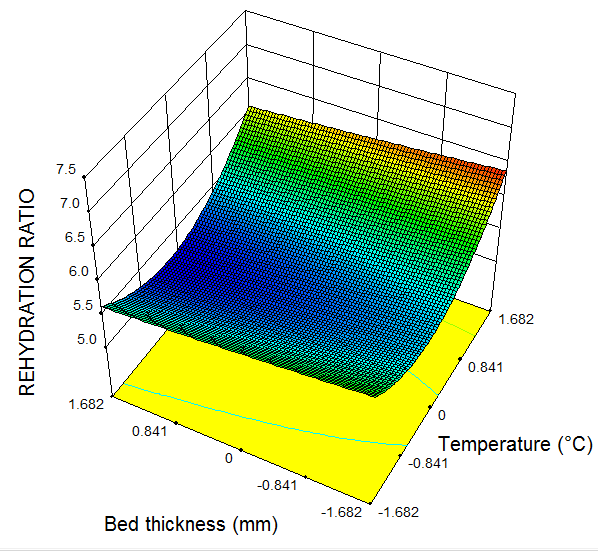


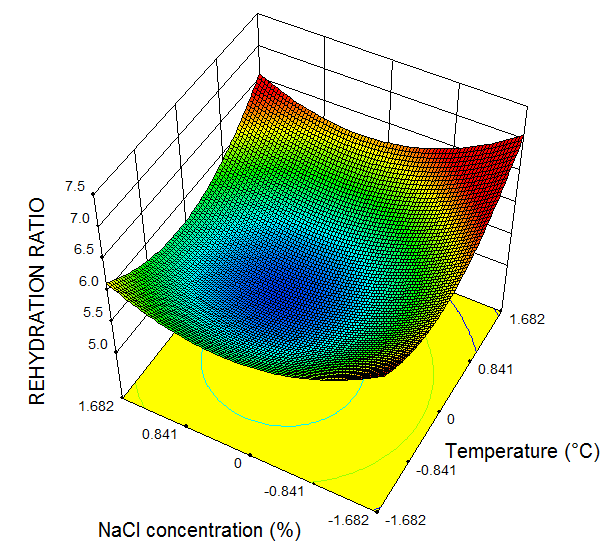


**Fig S3 :**Response surface plots for the effect of process variables on  Rehydration ratio of dried onion slices

| 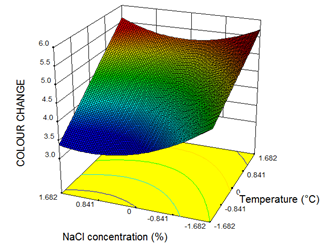 | 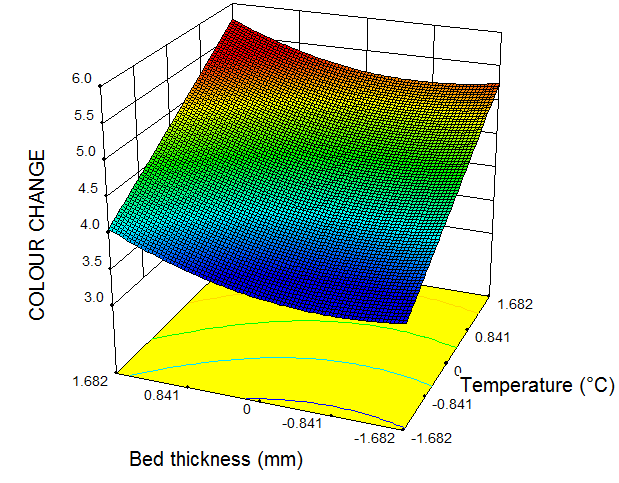 |
| --- | --- |
| 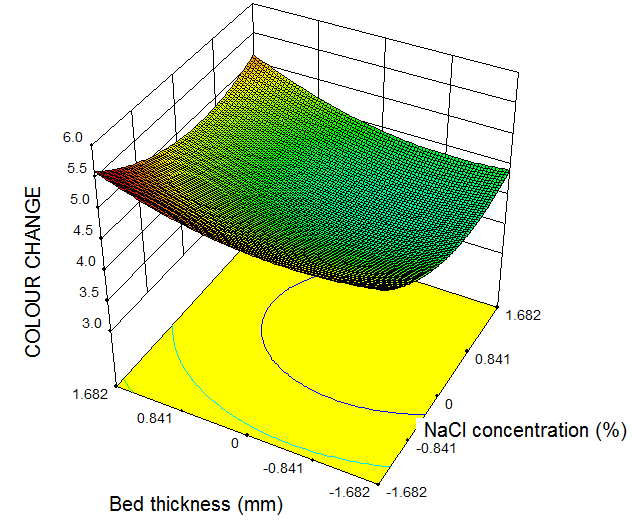 |  |

**Fig S4:  Response surface plots for the effect of process variables on colour  change of dried onion slices**

| 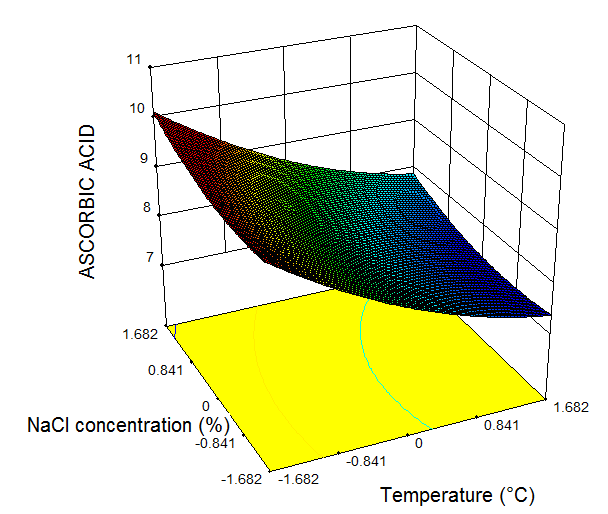 | 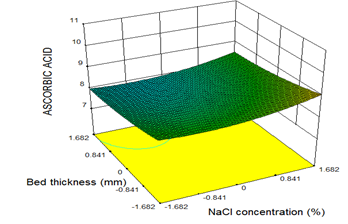 |
| --- | --- |
| 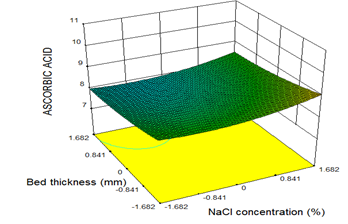  **Fig S5:  Response surface plots for the effect of process variables on  Ascorbic acid of dried onion slices** |  |

| 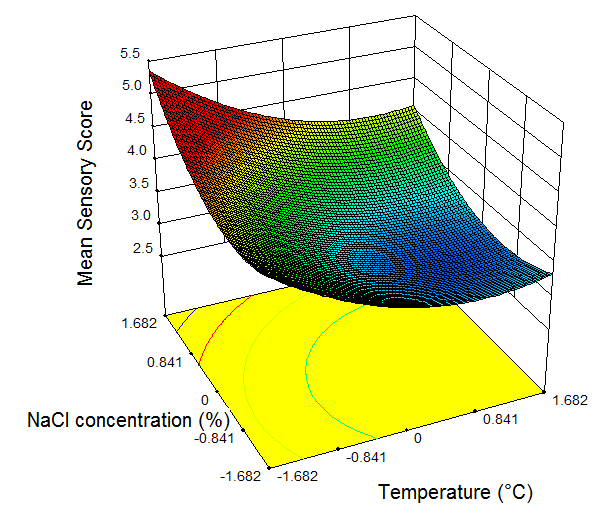 | 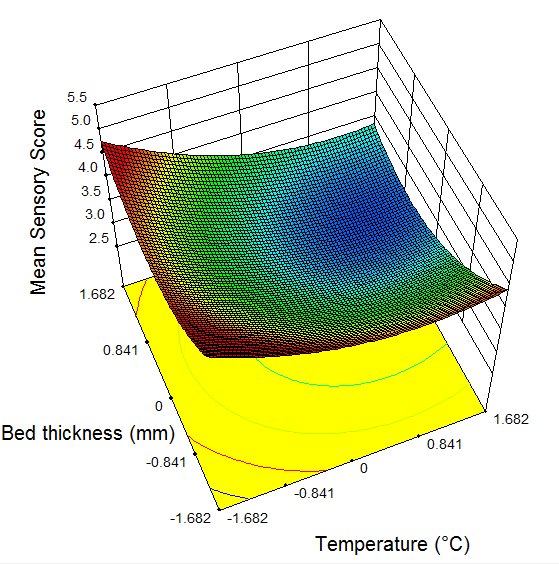 |
| --- | --- |
| 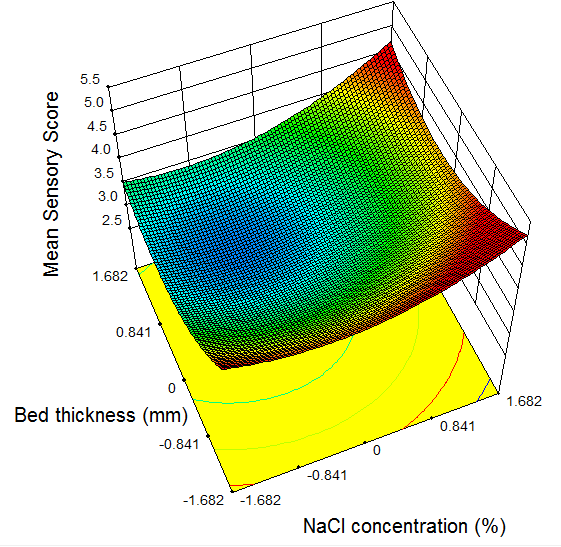 |  |

**Fig S6:  Response surface plots for the effect of process variables on mean  sensory score of dried onion slices**

|  |  |
| --- | --- |
|  |  |
|  |  |
|  |  |
|  |  |

**Fig S7: Linear variable graphs for all significant response**
